# Non-linear optical imaging of atherosclerotic plaques in the context of SIV and HIV infection prominently detects crystalline cholesterol esters

**DOI:** 10.1371/journal.pone.0251599

**Published:** 2021-05-13

**Authors:** Min Hi Park, Jeffrey L. Suhalim, Firas Elmastour, Santu K. Singha, Tadashi Imafuku, Ramanathan Venkatnarayan, Anette Christ, Alena Grebe, Sarah A. Oppelt, Dmitri Sviridov, Michael Bukrinsky, Eicke Latz, Eric O. Potma, Michael L. Fitzgerald

**Affiliations:** 1 Lipid Metabolism, Center for Computational and Integrative Biology, Massachusetts General Hospital, Harvard Medical School, Boston, Massachusetts, United States of America; 2 Department of Korean Medical Science, School of Korean Medicine, Pusan National University, Yangsan, Republic of Korea; 3 Department of Chemistry, Natural Sciences II, University of California, Irvine, California, United States of America; 4 University of Massachusetts Medical Center, Worchester, Massachusetts, United States of America; 5 Baker Heart and Diabetes Institute, Melbourne, VIC, Australia; 6 Department of Microbiology, Immunology and Tropical Medicine, School of Medicine and Health Sciences, The George Washington University, Washington, District of Columbia, United States of America; Maastricht University, NETHERLANDS

## Abstract

Chronic HIV infection may exacerbate atherosclerotic vascular disease, which at advanced stages presents as necrotic plaques rich in crystalline cholesterol. Such lesions can catastrophically rupture precipitating myocardial infarct and stroke, now important causes of mortality in those living with HIV. However, in this population little is known about plaque structure relative to crystalline content and its chemical composition. Here, we first interrogated plaque crystal structure and composition in atherosclerotic SIV-infected macaques using non-linear optical microscopy. By stimulated Raman scattering and second harmonic generation approaches both amorphous and crystalline plaque lipid was detected and the crystal spectral profile indicated a cholesterol ester (CE) dominated composition. Versus controls, SIV^+^ samples had a greater number of cholesterol crystals (CCs), with the difference, in part, accounted for by crystals of a smaller length. Given the ester finding, we profiled HIV^+^ plaques and also observed a CE crystalline spectral signature. We further profiled plaques from *Ldlr*^*-/-*^ mice fed a high fat diet, and likewise, found CE-dominate crystals. Finally, macrophage exposure to CCs or AcLDL induced auto-fluorescent puncta that co-stained with the LC3B autophagy sensor. In aggregate, we show that atheromatous plaques from mice, macaques and humans, display necrotic cores dominated by esterified CCs, and that plaque macrophages may induce autophagic vesicle formation upon encountering CCs. These findings help inform our knowledge of plaque core lipid evolution and how the process may incite systemic inflammation.

## Introduction

The AIDS pandemic erupted nearly four decades ago with the causative agent soon found to be uncontrolled HIV replication [1]. Severe viremia destroys host immune defenses and is followed by opportunistic infections, cancers and eventual death [2, 3]. Moreover, before the era of combined antiretroviral therapy (cART), cardiovascular pathologies including myocardial lesions and ventricular dysfunction were found to be AIDS related sequelae [4–7]. With early cART treatment, cardiovascular disease (CVD) complications evolved to more chronic metabolic conditions including dyslipidemia and lipodystrophy. Hypertriglyceridemia was particularly prominent in those treated with early protease inhibitor regimens [8, 9]. Modern cART has less off-target metabolic effects, but our, and others, work indicates that viral specific effects on lipid metabolism and CVD risk persist [10]. At the cellular level, the HIV Nef protein was found to modulate macrophage lipid trafficking by down-regulating the ABCA1 cholesterol efflux transporter [11, 12]. At the host level, infection status and viral load are associated with altered HDL structure and cholesterol acceptor function [13, 14]. Moreover, in people living with HIV, a state of chronic activation of the host immune system has been described, even when viral replication is suppressed, as in elite controllers, or by cART [15]. In aggregate, these observations suggest that chronic HIV disease is associated with increased atherosclerotic CVD risk beyond that accounted for by classic Framingham risk factors.

1.2 million people live in the US with HIV, while globally 35 million are infected, with 2 million new infections a year (US CDC.Gov). Hence, the finding that HIV infection may accelerate atherosclerosis has impact on the vascular complications management in this large and now long-lived cohort of individuals [16, 17]. Moreover, mechanistic understanding of how viremia accelerates atherosclerosis is of significant interest since recent evidence has shown that dysregulation of the innate immune system also drives plaque progression and CVD events. We have found that activation of the NLRP3 inflammasome, a cytoplasmic multi-protein complex that monitors host defense and lysosomal integrity is pro-atherosclerotic in mice [18]. Inflammasome activation leads to IL-1β secretion through a caspase 1 dependent proteolytic cleavage of the precursor IL-1β protein. Most excitingly, the recent results of the CANTOS trial indicate that anti-IL-1β antibody treatment added to a statin regimen significantly reduces CVD event risk [19]. However, how elevated IL-1β relates to CVD risk remains unclear. In mice, and in *ex-vivo* plaque cultures, we have found that crystalline cholesterol (CC) can induce IL-1β secretion by activating the inflammasome [20]. Moreover, in mice treated with β-cyclodextrin, an agent that reduces cholesterol crystals (CCs), progression of atherosclerosis can be blunted and even reversed [21].

These results are of interest since plaques rich in lipid and macrophages can rapidly progress to a hemodynamically vulnerable state with thin caps and necrotic cores [22]. HIV status has been associated with a greater burden of such vulnerable plaques as determined by CT-PET imaging [23, 24]. Hence, here we sought to further understand plaque structure in the context of HIV infection with the goal of determining CC burden and composition. As a discovery tool, we applied non-linear optical microscopy techniques. By this approach label-free contrast is generated through a nonlinear interaction between the light and the sample, such that incident photons preferentially stimulate Raman vibrations in the carbon-hydrogen bonds of the molecule of interest. Here, stimulated Raman scattering (SRS) and coherent anti-stokes Raman scattering (CARS) were utilized to characterize both amorphous and crystalline plaque lipid [25–27], and second harmonic generation (SHG) was used to confirm the crystalline state of the material deposits found in the plaques. Our major finding is that plaque composition in a model of SIV-infected macaque atherosclerosis and in coronary arteries from HIV+ individuals and from *Ldlr*^*-/-*^ mice all had a composition of CC in the esterified form. Moreover, our work suggests that macrophages upon encountering CCs induce autophagic vesicle formation. These finding helps clarify the pools of cholesterol that are prone to crystallization and help inform how CCs may trigger inflammasome activity and downstream IL-1β secretion.

## Materials and methods

### SIV Macaque atherosclerosis model

Descending aorta specimens were derived from a study of the role of SIV infection in accelerating atherosclerosis in the Macaque as published previously [28]. In brief, young adult male rhesus macaques (Macaca mulatta) were socially housed at the New England Primate Research Center in accordance with standards of the Association for Assessment and Accreditation of Laboratory Animal Care, and under the Harvard Medical School’s Animal Care and Use Committee approval (protocol # 04451). They were maintained on standard ad libitum normal diet (#5038, LabDiet, St. Louis, MO, USA), and then switch to an atherogenic diet ad libitum [diet no. 57JI; LabDiet, St Louis, MO, USA; total fat (39.35% of energy), saturated fat (13.78% of energy), cholesterol (1% of diet)] for 6 months, after which a subset of animals were intravenously inoculated with SIVmac239, and continued on the diet. Daily health checks for wellbeing and minimization of discomfort were made by the veterinary care staff in accordance with humane care endpoint principals and of the Weatherall report. In particular, the animals were housed in a biolevel 2 biocontainment facility in enrichment cages. Environmental enrichment was provided daily and included social interaction, manipulada, food treats, foraging feeding and puzzle feeders. Health checks were performed twice daily by animal care technicians to document findings and report abnormalities to the clinical veterinarian who provided follow up diagnostics and treatment if required. Analgesics were prescribed as needed by the clinical veterinarian and included meloxicam at 0.1–0.2 mg/kg and buprenorphine at 0.005–0.02 mg/kg. At study end, under isoflurane anesthesia, 9 weeks post infection, the animals were humanely euthanized by a pentobarbital-based overdose injection in accordance with the recommendations of the Panel on Euthanasia of the American Veterinary Medical Associations, and perfused with 4% paraformaldehyde in phosphate-buffered saline (PBS) at 95mmHg (approximate mean arterial pressure) for 20 min before necropsy. No animals were euthanized early due to human endpoints including occurrence of opportunistic infections, weight loss of > 10% BW from baseline, persistent diarrhea or other illnesses non-responsive to symptomatic treatment. The carotid arteries were collected bilaterally and samples were embedded in OCT media at the level of the bifurcation, and 5 μm unlabeled cryosections were imaged as described below.

### HIV^+^ LDA autopsy specimens

A protocol was developed and approved by the National Disease Research Interchange Tissue Bank to obtain autopsy specimens of the left descending coronary artery from HIV^+^ individuals (FIM2-001, Protocol-005). In brief, within 24 h of death, at autopsy, samples were dissected, fixed in 10% formalin, shipped on dry ice, and stored at -80°C till processed for embedding in OCT media. Embedded samples were used to produce 5 μm cryosections for label free imaging as described below. The MGH IRB determined that the NDRI protocol research activities did not meet the definition of human subject research since it involved no interventions with the living that would not otherwise have occurred, and no identifiable, or associable, data or information was obtained.

### Mouse atherosclerosis model

*Ldlr*^*-/-*^ mice on a C57/BL/6 background (Jackson Labs) were fed a high fat diet for 8 weeks (Harlan Teklad, TD 88137, 21% Fat, 0.2% cholesterol, 42% calories derived from anhydrous milkfat). Under Isoflurane anesthesia, a terminal bleed was taken by cardiac puncture, the vasculature was perfusion flushed with 10 ml of phosphate buffered saline. The heart and aortic arch were dissected, fixed in 10% formalin, and embedded in OCT media, and aortic 5 μm cryosections proximal to the aortic valve were obtained. Femur bone marrow was collected from the animals on the HFD for either 4 or 8 weeks, red blood cells were lysed (Sigma, Cat#R7757), adherent cells were removed by on overnight culture in differentiation media [DMEM, 10% FBS, 15% L929 conditioned cell media, penicillin/streptomycin (Cellgro Cat#30-001-C1)] and immature bone marrow derived macrophages were further cultured in differentiation media for 5 days before confocal imaging analysis. The animal work was reviewed and approved by the MGH IACUC.

### Cell culture models of cholesterol accumulation

RAW264.7 or J774A.1 mouse macrophage cell lines were cultured in Dulbecco’s Modified Eagle’s Medium (DMEM) with 10% fetal bovine serum (FBS) and 0.5 μg/mL penicillin-streptomycin in 35 mm glass bottom culture dishes at 100,000 cells/dish. Cholesterol crystals were prepared as described and used to treat RAW264.7 cells (200 ng/ml, 24 h) [20]. J774A.1 cells were treated with AcLDL (40 μg/ml, 24 h). Detection for nuclei (Hoechst 33258, Sigma–Aldrich), lipid droplets, endoplasmic reticulum and autophagosomes (Nile Red, # N1142, ER-Tracker^™^ Red, # E34250, LC3B-RFP, # P36236 Invitrogen/ThermoFisher) were performed as described by the manufacturer.

### Raman micro-spectroscopy

Raman spectra were collected with a 50x objective lens at selected points in the tissue samples with a Raman microscope (InVia, Renishaw). The excitation wavelength was 532 nm and the spectral acquisition time was 1 s, with up to 10 averages to reduce noise.

### Nonlinear optical microscopy

SRS was used to visualize plaque and the lipid inclusions in a label free manner, as previously described [27]. In brief, unstained sections were imaged using a custom built inverted confocal microscope (Fluoview 300, Olympus) with a light source comprised of an optical parametric oscillator (Levante Emerald OPO, APE, Berlin) pumped by the second harmonic of a Nd:vanadate picosecond mode-locked laser (PicoTrain, High-Q). The OPO is tunable in the 750–960 nm range and provided the pump beam for the coherent Raman process, whereas the residual radiation from the Nd:vanadate laser at 1064 nm was used as the Stokes beam. The beams were overlapped in space and time and directed to the laser-scanning microscope. The average power of both the pump and Stokes beams was kept below 30 mW at the sample. Images (512x512 pixels) were collected at 1 frame/s, and up to 10 frames were averaged to reduce noise. The frequency difference between the pump and Stokes beams was tuned to the 2800–3100 cm^-1^ range, which corresponds to the energies of the stretching vibrations of CH_2_ groups. In this range, lipid rich regions give rise to strong signal contrast due to the greater density of these groups in lipids relative to other molecules such as nucleic acids and proteins. When collecting data in the SRS mode both amorphous and crystalline plaque lipids were detected and the composition of the crystals is determined by comparing their spectral signatures to that of pure crystal standards of cholesterol, cholesteryl linoleate, cholesteryl oleate. Hyperspectral data stacks were recorded automatically with the aid of a Python script, which acquired images in the 2825–3075 cm^-1^ range at 5 cm^-1^ intervals. Spectra were extracted at selected regions of interest and represented as one-dimensional plots, using a B-spline between spectral points to highlight the main features of vibrational spectra. To selectively image crystalline cholesterol, without detecting amorphous lipid, second harmonic generation microscopy was employed. In SHG imaging, contrast is uniquely sensitive to the non-centrosymmetric ordering of crystalline cholesterol, whereas non-crystalline lipid forms do not generate frequency doubling of impingent photons, and hence, no contrast is generated. Finally, for the macaque study, coherent anti-Stokes Raman scattering (CARS) was additionally used to define morphological landmarks in the plaque and vessel wall [25].

### Hyperspectral scans, crystal composition and burden assessment

High-resolution spectral maps were obtained as previously described [26]. In brief, an x by y area is scanned at consecutive wavenumbers (*ω*n), forming a 3D hyperspectral data cube (x,y,n) where each pixel in the x-y plane contains a vibrational spectrum. The spectral signature of crystalline and non-crystalline objects in the image area is then compared to the spectral references of free non-esterified cholesterol, cholesteryl linoleate, cholesteryl oleate and protein. Crystal burden was then assessed by two approaches in the Macaque samples. For the SHG images, binary images derived by Otsu’s method were subjected to watershed segmentation and overlapping objects were removed. Then the number and length of the crystalline objects in the resulting image was computed. Reflectance confocal microscopy was further used as previously described to measure overall plaque crystal burden, macrophage content and cellularity [20]. In particular, a Leica TCS SP5 II AOBS confocal laser-scanning microscope was employed where the detector and acousto-optical beam splitter were set to allow the detection of reflected laser light using a 475 nm excitation wavelength. Plaque CC content was quantified from 3–4 sections per macaque using Volocity Quantitation (PerkinElmer) and depicted as a ratio of total crystal reflection area to total plaque area. Plaque cellularity and macrophage content were assessed using standard fluorescent detection of DAPI stained nuclei and anti-CD68 macrophage detection. Investigators performing the histological analyses were blinded to the infection status of the animal.

### Statistics

Statistical tests utilized were the Mann-Whitney U test and two sample (unpaired) t-tests. A p value <0.05 was considered significant.

## Results

### CC composition and burden in SIV infection

**Fig 1:**
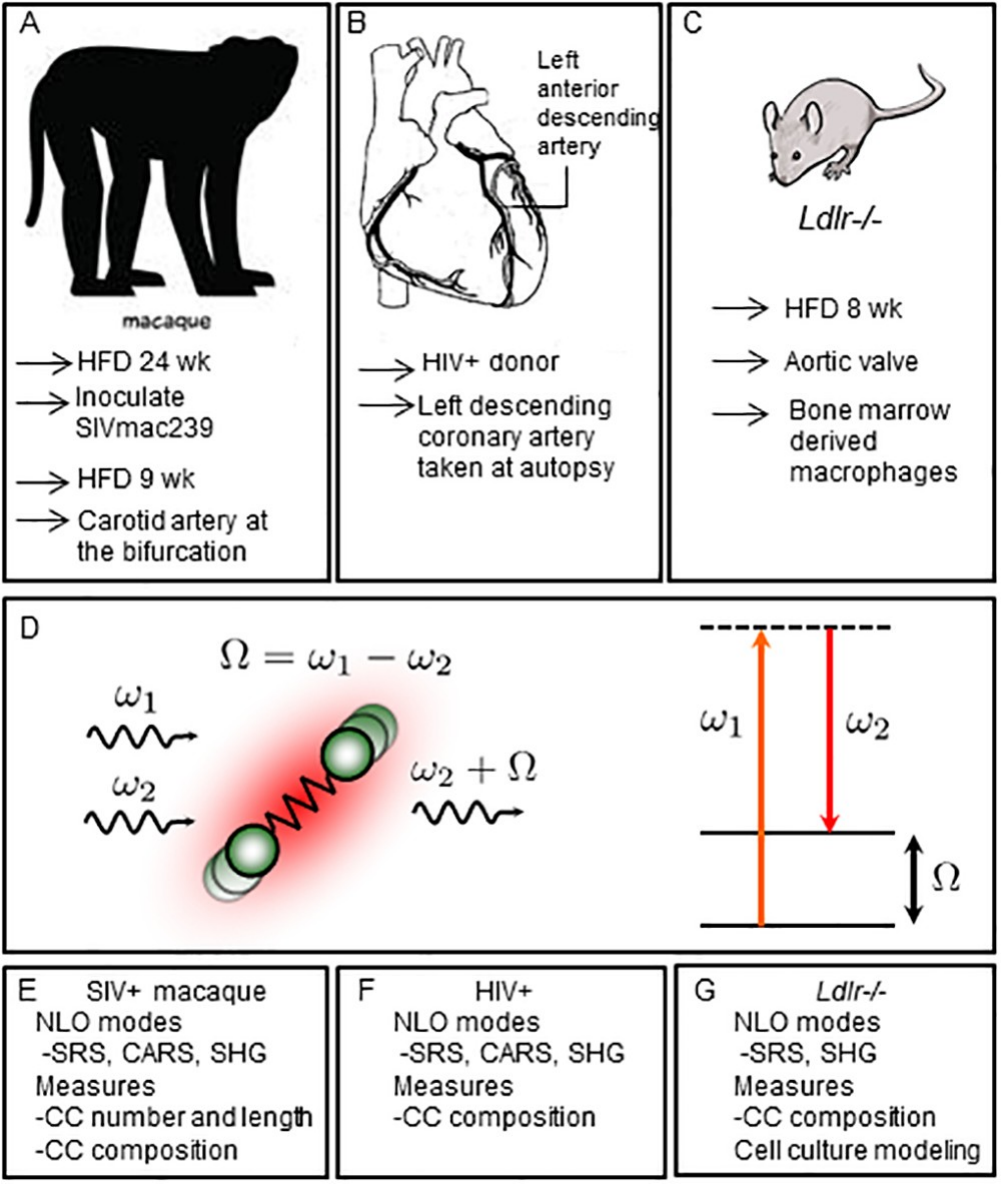
Atherosclerotic models and plaque imaging by stimulated Raman loss. Plaque samples were derived from three sources, (A) a study of male Macaques on a high fat diet for 24 weeks, inoculated with SIVmac239, or not, and kept on diet another 9 weeks before sacrifice and analysis, (B) HIV^+^ autopsy samples of the left anterior descending coronary artery, (C) a study of *Ldlr*^*-/-*^ mice fed a high fat diet for 8 weeks. (D) Diagram of the mechanism of the stimulated Raman loss process, where ω_1_ and ω_2_ are the frequencies of the incoming photons, and Ω corresponds to the frequency of the molecular vibration. (E-F) Imaging modalities used and measures taken in the models.

**Fig 2:**
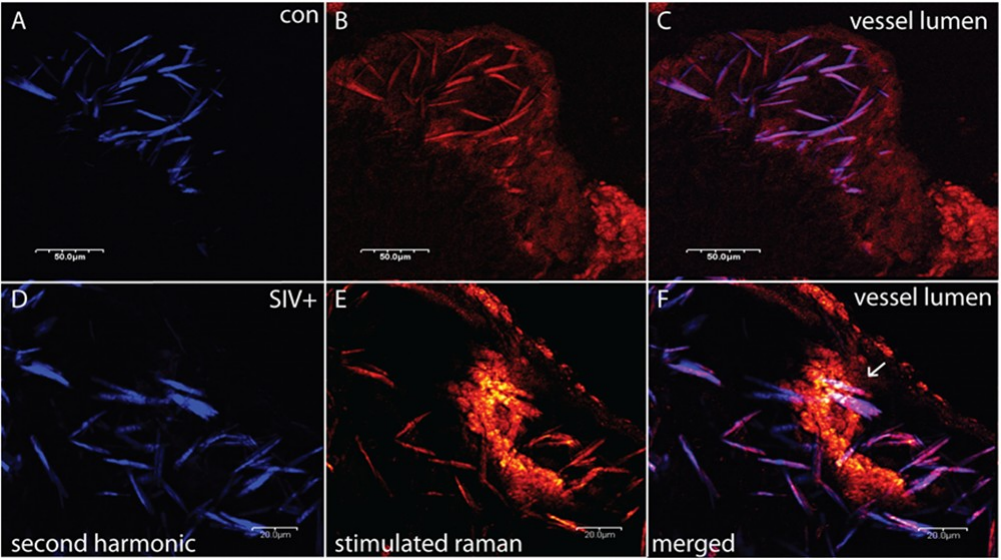
Control and SIV^+^ plaques display needle-like CCs as detected by SRS and SHG imaging. Control plaque, top three panels, SIV^+^, bottom three, with SHG signal shown in (A & D, blue) SRS signal (B & E, orange) and composites (C & F). Arrow in F points to a macrophage attempting to phagocytose a CC. Scale bars are in micrometers (μm), and vessel lumen is denoted.

How retroviral infection may affect plaque cholesterol composition was investigated using samples of aortic atherosclerotic plaques from a rhesus macaque study. As described previously, young males received a diet high in fat and cholesterol for 6 months, then a subset of these were inoculated with SIVmac239, and all were further maintained on diet 9 weeks post-infection till sacrificed (Fig 1A) [28]. Unstained carotid artery cryosections were first imaged by SRS microscopy. As diagramed in Fig 1D, this approach employs a two-photon light-matter interaction to stimulate bond vibrations in the sample that provide the contrast in the image. Importantly, this optical process has specificity for distinct molecular classes. For lipid-rich molecules, CH_2_ group vibrations dominate in the high-energy vibrational range and SRS imaging detects both amorphous and crystalline lipid. Fig 2 shows an advanced plaque of an uninfected animal (panels A-C), while the lower panels are from a SIV^+^ animal (D-F). The SRS channel is colored red (Fig 2, panels B & E), while data captured in the SHG mode, where image contrast is uniquely sensitive to the non-centrosymmetric ordering of crystalline cholesterol, is shown in blue (Fig 2, panels A & D), with the merged images shown in panels C & F. The control plaque has an advanced morphology with a large core of CCs, a thin proteinaceous cap, and interestingly, a large buildup of amorphous lipid rich tissue (right shoulder region). The SIV^+^ sample, shown at a higher magnification, also has a bed of sub-endothelial cholesterol crystals. Importantly, also seen in this space is a lipid rich macrophage foam cell attempting to phagocytose one of the CCs (Fig 2, panels E & F, note the low contrast polymorphic nuclei due to its lower lipid content).

**Fig 3:**
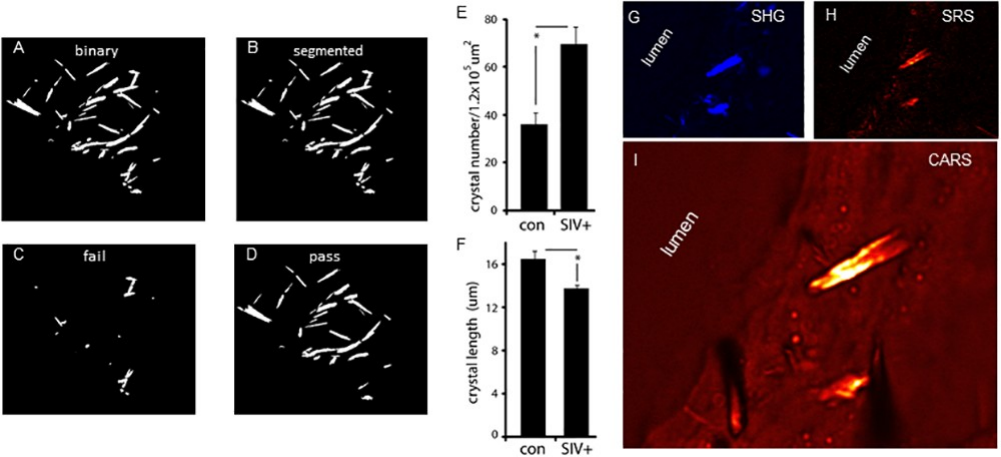
Counts of CCs number and length by Otsu’s method. (A) binary rendering of the SHG control plaque image shown in Fig 2A–2C, (B) after segmentation, (C) failed overlapping objects (D) passed independent objects. (E) Graph of CC number per unit area of control and SIV^+^ plaques, (F) Graph of CC length of control and SIV^+^ plaques. For E & F a total of 973 objects were counted from 5 animals (con-2, Siv^+^-3, 3–4 images/animal, p value <0.05 for E, and <0.0001 for F, by paired t- test). (G-I) A SIV+ plaque showing CCs in the luminal endothelial layer using SHG (G), SRS (H), and CARS (I).

We next developed an image processing protocol allowing us to count and measure the length of the plaque crystalline needles in the macaque samples by Otsu’s method and Watershed segmentation [29]. The binary and segmented results of the SHG control plaque image (Fig 2A) are shown in Fig 3 (panels A & B). Upon removing needle-shaped objects with large overlapping regions (Fig 3C) the binary pass image was generated and quantified (Fig 3D). A total of 973 needle structures were counted for the control and SIV^+^ images, and as expressed per plaque area, the SIV^+^ samples had more crystals (Fig 3E). However, in part, this increase was due to the SIV^+^ plaques having needle structures of significantly reduced length (Fig 3F). Moreover, by confocal reflectance microscopy as a second method assessing plaque crystal burden, a significant difference in plaque crystal burden was not found (S1 Fig). Interestingly, however, instances of these smaller needles of the SIV^+^ samples were found within the endothelial layer close to, if not piercing, the luminal wall (Fig 3, panels G-I).

**Fig 4:**
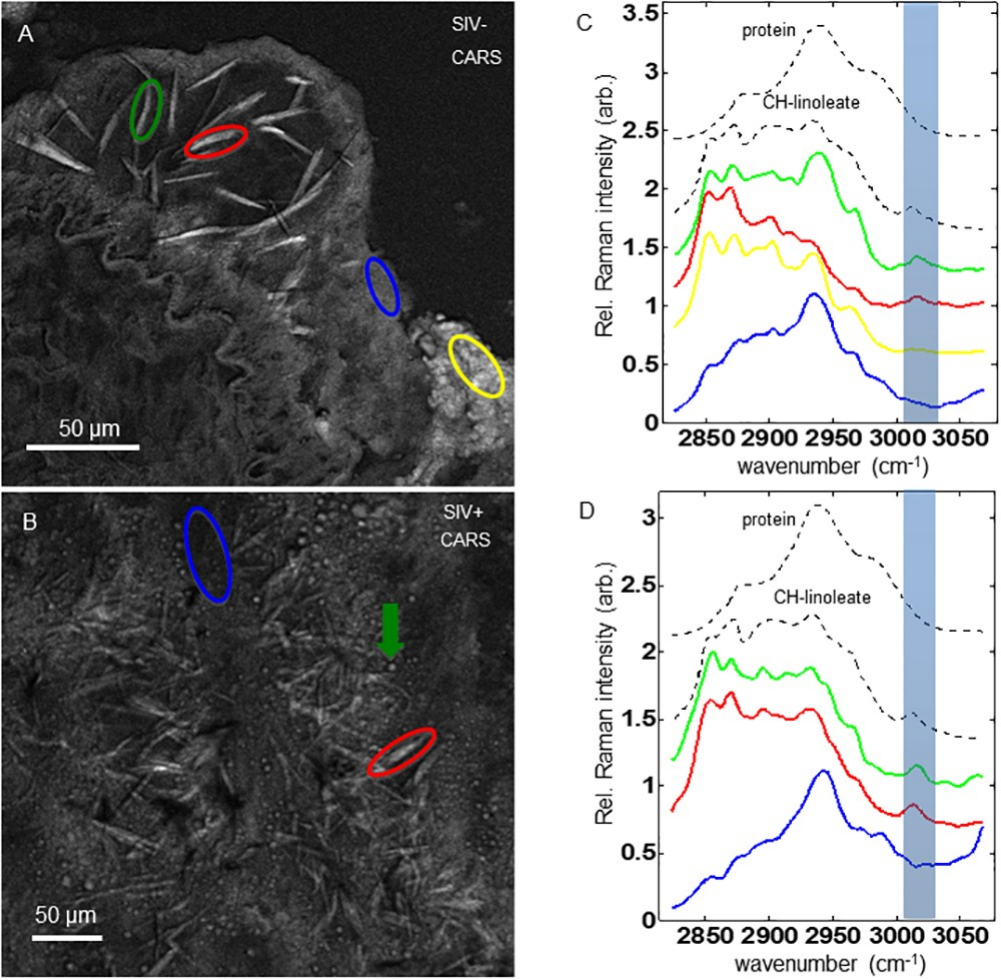
Hyperspectral scans of SIV^+^, and control, plaques detect a CE dominate signature. (A & C) spectral traces for two needle objects (green and red ovals), a protein rich area of the shoulder region (blue) and an amorphous lipid rich shoulder region (yellow) are shown for the control plaque. (B & D) SRS spectral traces for a needle object (red), a protein region (blue), and an amorphous lipid droplet (green arrow) of a SIV^+^ plaque. The top two dashed black traces in C & D represent protein and cholesteryl-linoleate reference SRS spectra, respectively. Shaded spectral band indicates the spectral response of unsaturated acyl chains.

Employing SRS microscopy’s second powerful feature, hyperspectral scans, we generated data cubes for the control and a SIV^+^ plaque (Fig 4, panels A & B). In the control plaque core region, we then extracted data from two interest regions encompassing needle structures (red and green circles). In the shoulder area, a low contrast, proteinaceous region (blue), and a high contrast amorphous lipid rich region (yellow) were also selected. Graphed at the right is the spectral intensity vs. wavelength traces of these objects (Fig 4C, colored), and they are compared to spectral standards for pure protein (top dotted trace) and crystalline cholesteryl-linoleate (second dotted trace, Fig 4C). Likewise, in the SIV^+^ sample we derived traces for a needle shaped structure (Fig 4B, red circle), as well as a lipid rich amorphous droplet (Fig 4B, green arrow) and a low contrast protein region (Fig 4B, blue circle). Significantly, the spectral traces of both control and SIV^+^ crystalline needles showed a peak at approximately 3010 cm^-1^ which is characteristic of the C = C-H group, and an indicator of unsaturated acyl chains found in cholesterol esters such as cholesteryl linoleate (Fig 4C & 4D, blue highlighted region of graphs).

### CC composition in HIV infection

**Fig 5:**
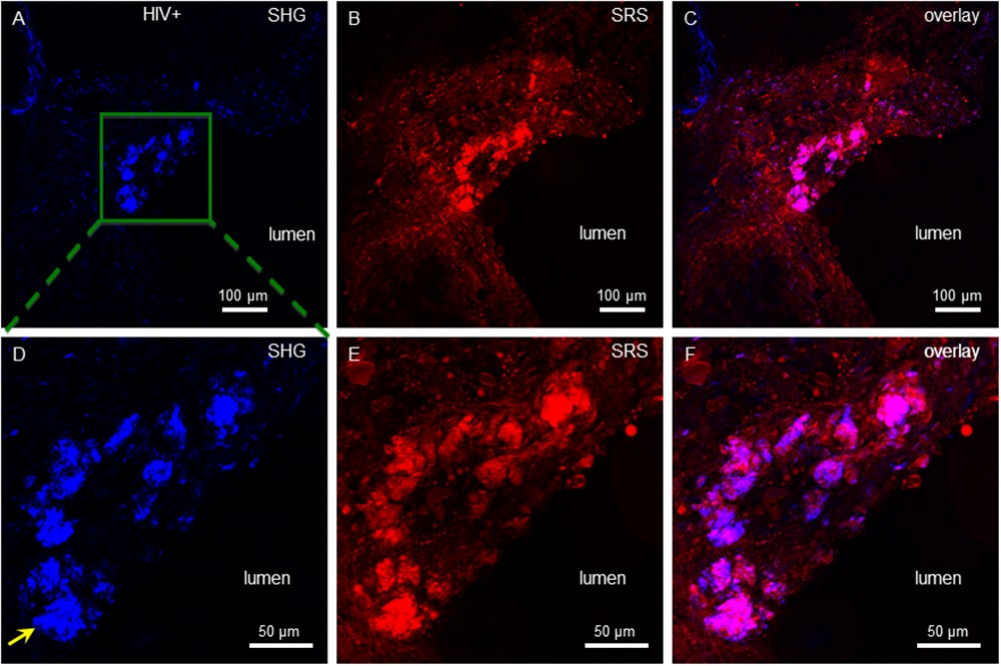
A lipid rich HIV^+^ coronary artery plaque with aggregated cuboidal CCs. SHG (A), SRS (B) and overlay (C) images of a lipid rich plaque. Panels (D), (E) and (F) show the same plaque at higher magnification, corresponding to the area enclosed by the green box in (A). SRS images were recorded at 2845 cm^-1^. Magenta indicates regions of high overlap in the overlay images. Vessel lumen of the left descending artery and magnification scales are indicated, and yellow arrow in panel D denotes cuboidal feature shown in higher detail in Fig 7D.

**Fig 6:**
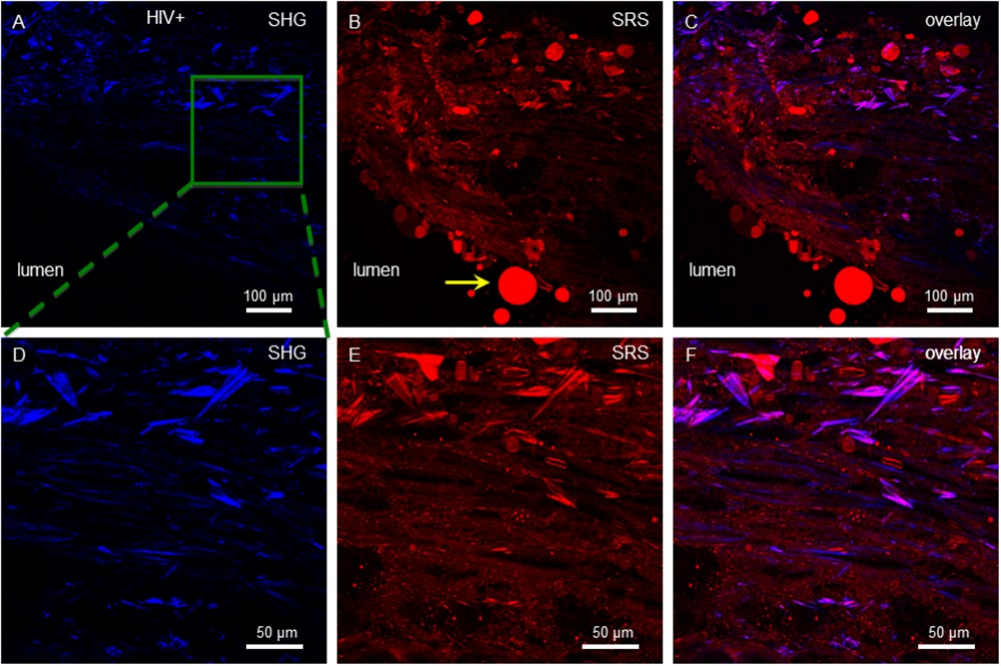
Lipid rich plaque from HIV^+^ coronary artery showing needle CCs. SHG (A), SRS (B) and overlay (C) images of a lipid rich plaque. Panels (D), (E) and (F) show the same plaque at higher magnification, corresponding to the area enclosed by the green box in (A). SRS images were recorded at 2845 cm^-1^. Magenta indicates regions of high overlap in the overlay images. Vessel lumen of the left descending artery and magnification scales are indicated, and yellow arrow in panel B indicates fat droplet in vessel lumen derived from extravascular adipose tissue.

**Fig 7:**
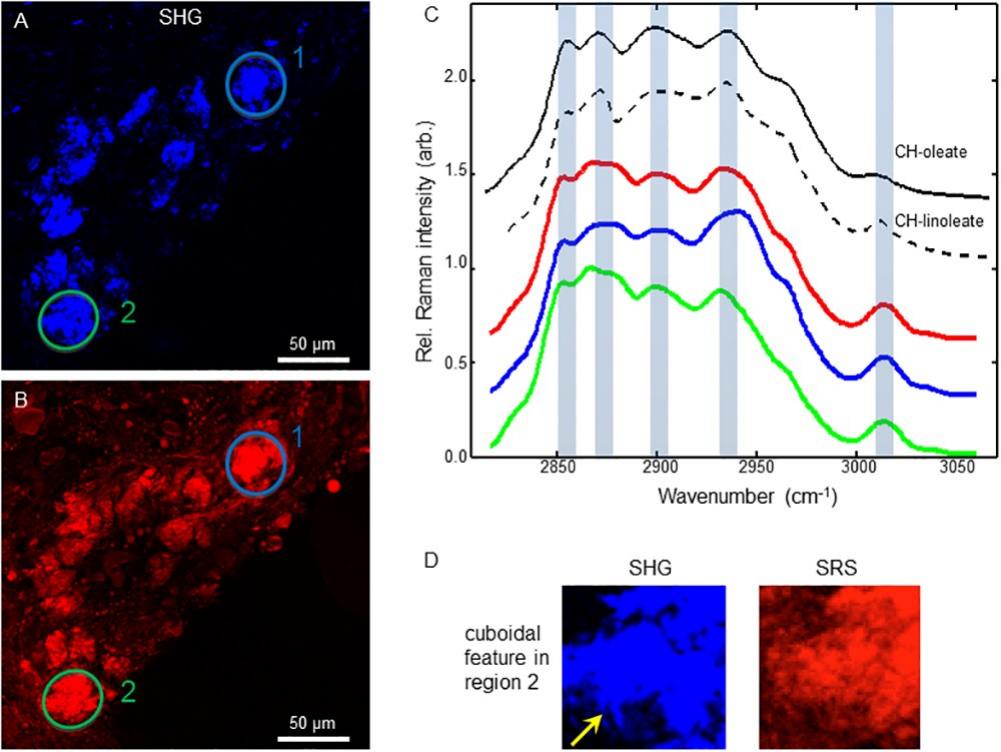
Hyperspectral scan of HIV^+^ plaque aggregated cuboidal CCs detect a CE dominate signature. Left panels show the SHG (A, top) and SRS (B, bottom) with two regions of interest denoted (green and blue circles). Graphed right (C) are the SRS spectra of the individual objects (green and blue lines) and their average (red). The top black solid and dashed traces represent the SRS spectra of cholesteryl oleate and linoleate standards, respectively. Detail of green region 2 is shown in (D) for the SHG and SRS channels, and the cuboidal structure is indicated by the yellow arrow.

**Fig 8:**
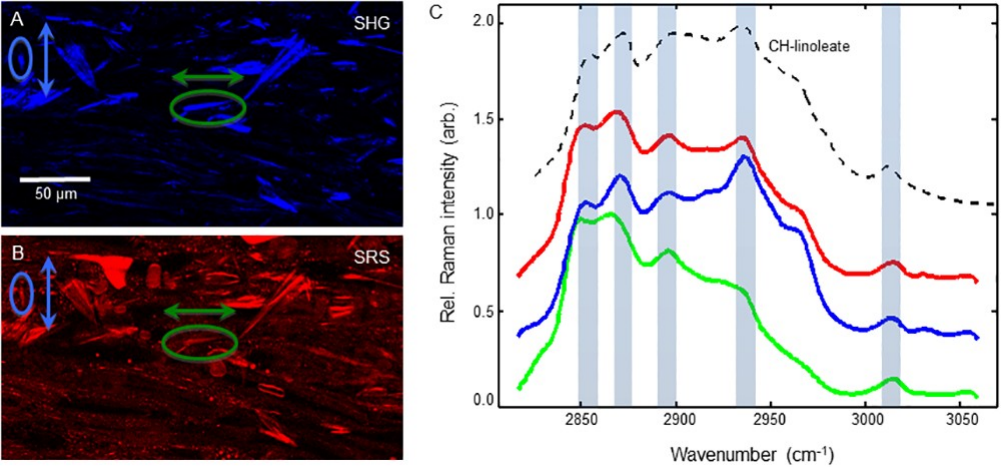
Hyperspectral scan of HIV^+^ plaque needle CCs detect a CE dominate signature. Left panels show the SHG (A, top) and SRS (B, bottom) with two object regions denoted (green and blue ovals with arrows indicating relative polarization direction). Graphed right (C) are the spectral traces of the individual objects (green and blue lines), their average (red), with the top black dashed trace showing the cholesteryl linoleate reference SRS spectrum.

Having found crystalline cholesterol ester needles in the macaque model of SIV infection, we analyzed atherosclerotic plaques from HIV^+^ autopsy samples. A NDRI protocol was developed to obtain the left descending coronary artery of HIV^+^ donors post-mortem (Fig 1B & methods). Similar to the macaque model, SHG and SRS imaging of cryosections showed lipid rich plaques that had significant SHG signal indicating the presence of crystalline lipids (Fig 5). However, a more aggregated morphology with occasional cuboidal features dominated (yellow arrow, Figs 5D, and 7D in more detail), although needle structures were also found (Fig 6), so hyperspectral data sets of both morphologies were obtained. In the aggregated crystalline material, including that with a cuboidal feature, spectral signatures of two interest regions were analyzed, and the scans were again consistent with a cholesterol-ester dominated composition (Fig 7). Likewise, spectral analysis of needle-shaped crystals also indicated a cholesterol-ester dominated composition (Fig 8).

### CC composition in atherosclerotic *Ldlr*^*-/-*^ mice

**Fig 9:**
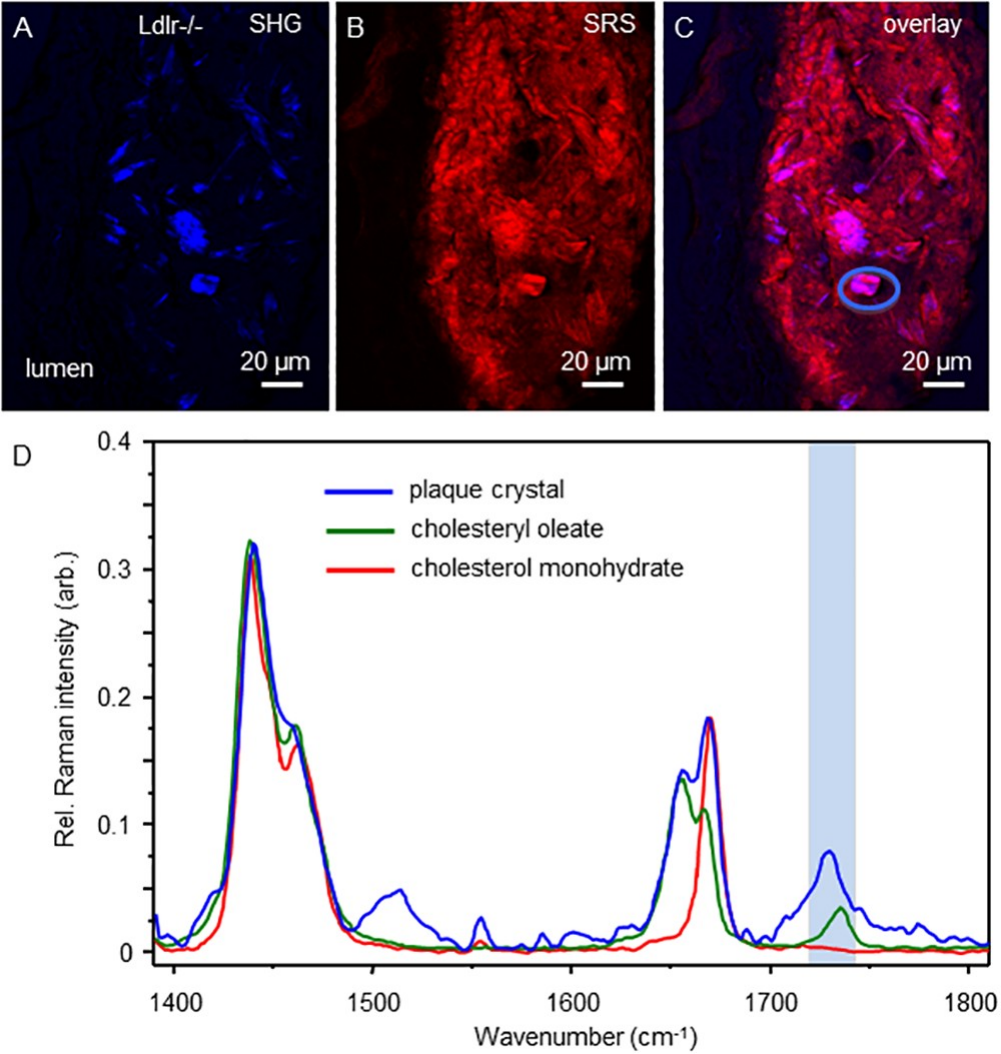
Hyperspectral scan of a *Ldlr*^*-/-*^ plaque cuboidal CC detects a CE dominate signature. Top panels (A-C) show SHG, SRS and composite image of a lipid rich aorta plaque with a cuboidal crystalline region denoted (C, blue oval). Graphed below (D) is the spectral traces of the CC object (blue line) as well as standard traces of cholesteryl oleate (green) and free cholesterol monohydrate (red). The finger print region of the traces in the graph is highlighted (blue rectangle).

In atherosclerotic mouse models, in addition to CE dominate crystals, free non-esterified cholesterol crystals are also found, particularly in the nephrectomized *ApoE*^*-/-*^ model [25]. Hence, we further analyzed plaques from *Ldlr*^*-/-*^ mice fed a high fat western diet for 8 weeks (Fig 1C). Using conventional Raman micro-spectroscopy in the finger print region to examine an object with cuboidal morphology, the data was again most consistent with a cholesterol-ester dominated composition as a strong peak at 1740 cm^-1^ region was seen in the plaque object and the cholesteryl-oleate standard but not the cholesterol monohydrate standard (Fig 9).

### Macrophages induce autophagic vesicles in response to CCs and AcLDL

**Fig 10:**
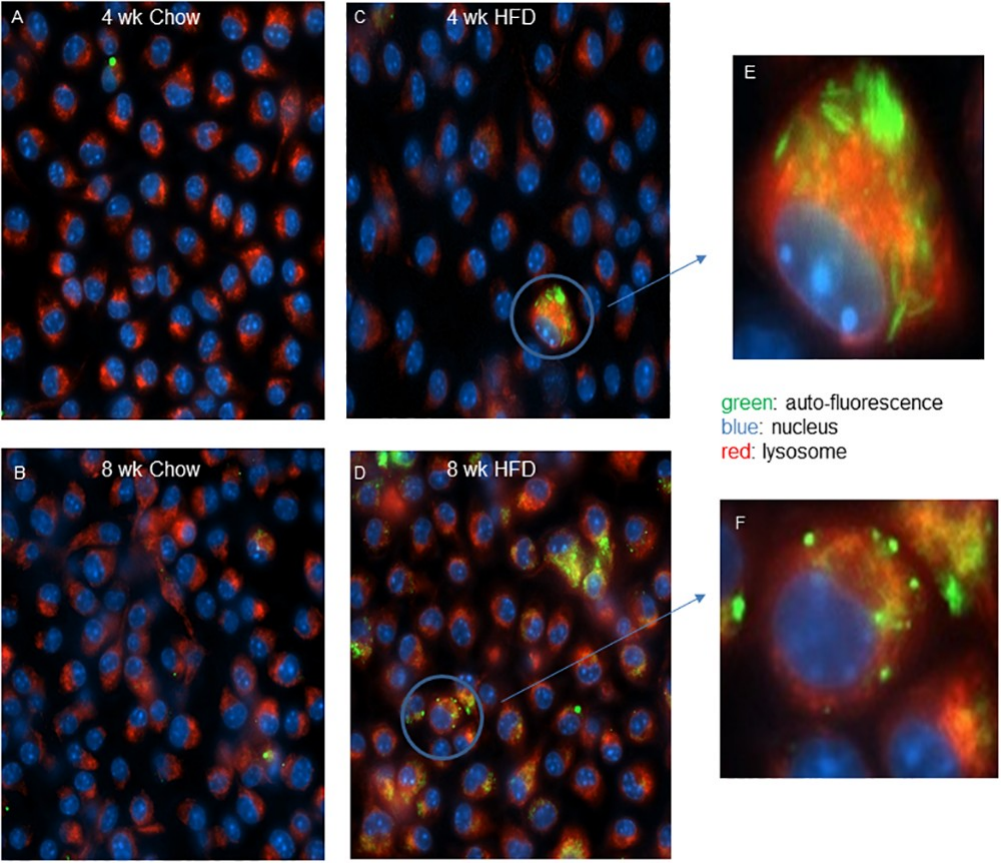
*Ldlr*^*-/-*^ bone marrow derived macrophages from HFD fed *Ldlr*^*-/-*^ mice display a lipid engorged phenotype. *Ldlr*^*-/-*^ mice were fed a chow or a HFD for 4 (panel A & C) or 8 weeks (B & D) and the bone marrow derived macrophages cultured for 5 days were imaged for auto-fluorescence (green) and counterstained for nuclei (blue) and lysosome (red). Circled area in C & D are shown in more detail in E and F, respectively.

**Fig 11:**
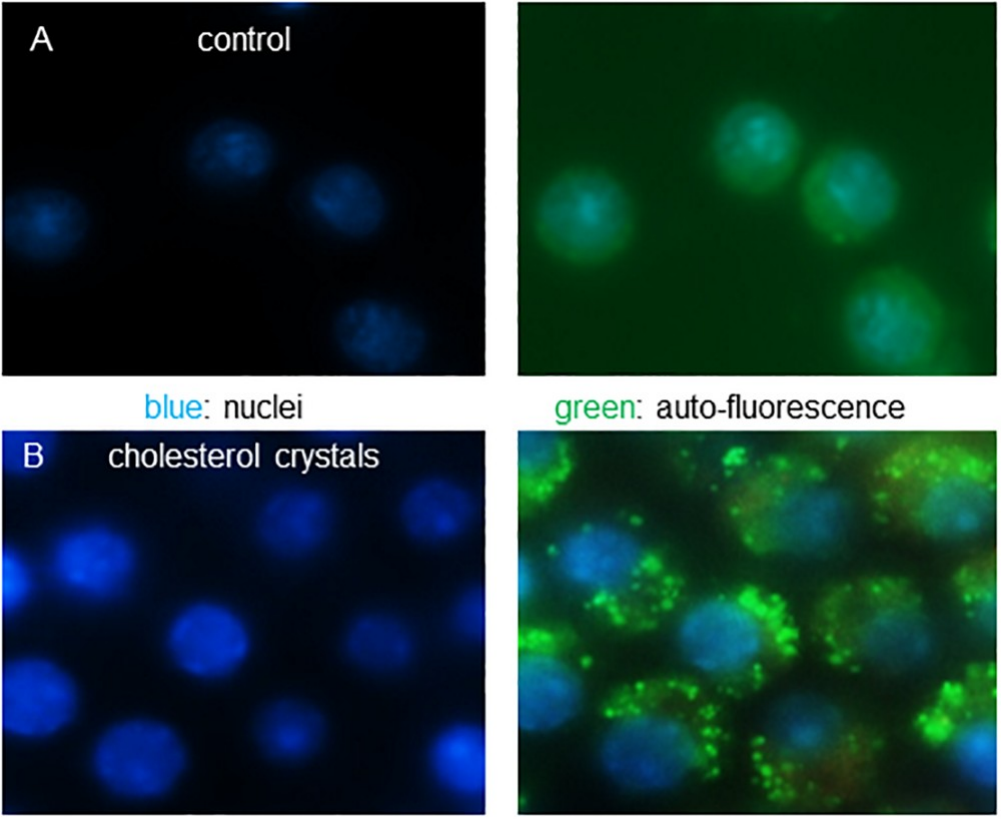
Cholesterol crystal uptake by J774A.1 macrophages induces auto-fluorescent puncta. Cholesterol crystals (200 μg/ml) were added to cultured J774A.1 macrophage for 24 h and compared to vehicle treated cultures. A, Control treated cells, B, cholesterol crystal treated cell with left panels showing stained nuclei, and right panels showing auto-fluorescence and nuclei.

**Fig 12:**
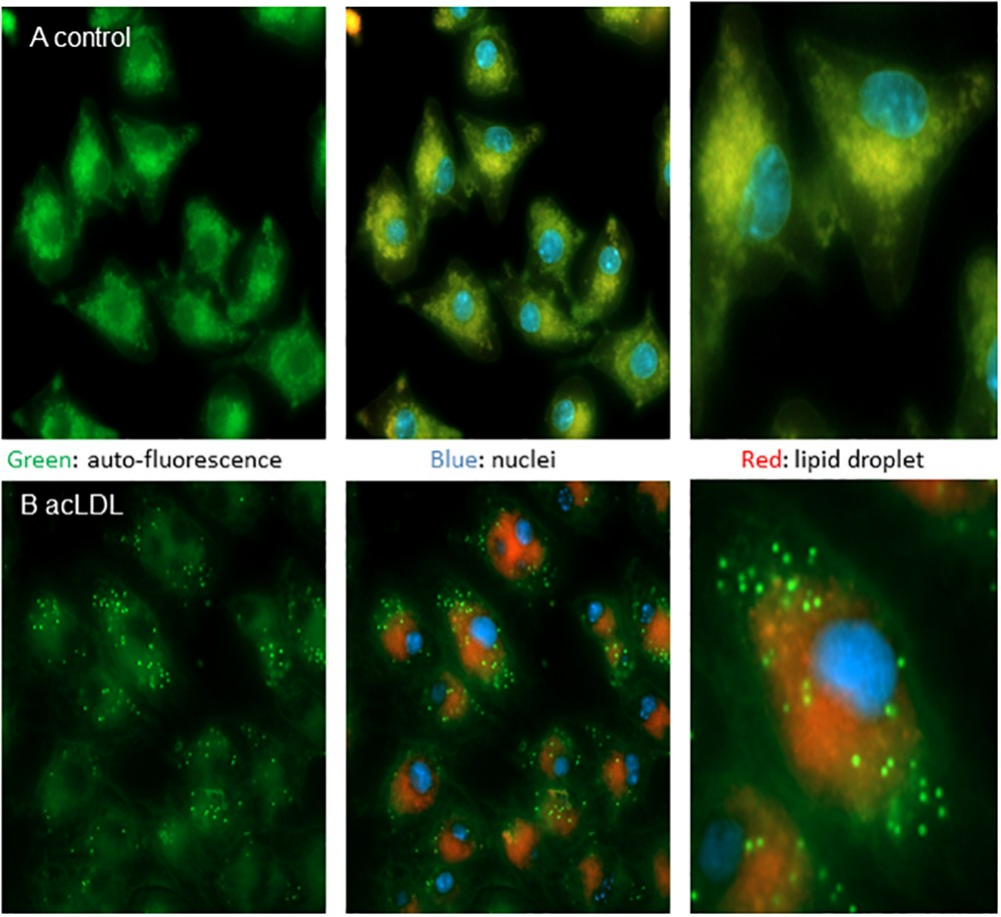
AcLDL uptake by J774A.1 macrophages induces auto-fluorescent puncta distinct from the lipid droplet. Vehicle (A, top panels), or AcLDL (B, bottom panels, 40 μg/ml), was added to cultures for 24 h and cells were imaged for auto-fluorescence (green) nuclei (blue) and lipid droplets (red) with right panels showing selected cells in more detail from the center panels.

**Fig 13:**
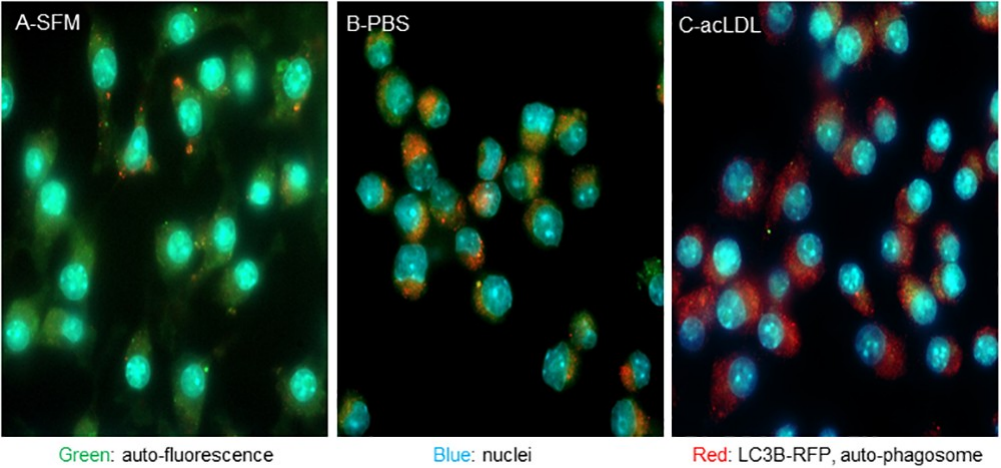
AcLDL induced auto-fluorescence puncta co-localize with auto-phagosome LC3 activity. J774A.1 cells were transfected with LC3B-RFP, and treated with AcLDL (C, 40 μg/ml, 24 h), starved in PBS (B, positive control for autophagic induction, 2 h), or left in serum free media (A, negative control for autophagic induction), and imaged for auto-fluorescence shown in green, nuclei in blue, and LC3B-RFP distribution in red.

We have shown that brief high fat feeding can alter the epigenetic state of bone marrow myeloid stem cells [18]. Here we assessed if there was also evidence of altered lipid accumulation in cells derived from this compartment. We isolated and cultured bone marrow derived macrophages from *Ldlr*^*-/-*^ mice fed chow or a diet high in fat for 4 or 8 weeks. At 4 weeks, occasionally cells from the high fat fed mice were found that exhibited a lipid engorged phenotype as evidenced by increased auto-fluorescent lipid inclusions, with some of these structures potentially having a crystalline morphology (Fig 10C & 10E). At 8weeks of feeding a greater portion of the cells demonstrated auto-fluorescent structures, and some of these had distinct puncta that did not overlap with lyso-tracker staining of the lysosome (Fig 10D & 10F). Feeding Raw264.7 murine macrophages cholesterol crystals also strongly induced the auto-fluorescent punta (Fig 11), as did treatment of J774A.1 macrophages with AcLDL (Fig 12). Here again, the puncta did not significantly overlap the Nile red staining of lipid droplets. Finally, we tested in the AcLDL loaded J774A.1 macrophages if puncta overlapped with the autophagic marker LC3B, which was found to be the case indicating the puncta were autophagic vesicles (Fig 13).

## Discussion

Here we used advanced optical microscopy to image atherosclerotic plaques, assessing crystal burden and composition in two settings of retroviral infection, and in a mouse model of sterile diet driven inflammation. In male atherosclerotic macaques, SIV infection did not markedly increase overall cholesterol crystal burden. However, the SIV^+^ samples did display a greater number of smaller crystals. Additionally, the SIV^+^ samples showed evidence of cholesterol crystals piercing the endothelial layer and a macrophage foam cell attempting to phagocytose cholesterol crystals. Hyperspectral scans of both SIV^+^ and control plaques indicated the needle shaped crystals had a cholesterol-ester dominant signature. Atherosclerotic plaques in HIV^+^ coronary arteries displayed both cuboidal and needle crystals, and again both had a cholesterol ester dominate spectral signature, as did plaques from the high fat fed *Ldlr*^*-/-*^ mice. *Ex vivo*, bone marrow derived macrophages from the atherosclerotic *Ldlr*^*-/-*^ mice displayed a more lipid engorge phenotype, and cell line modeling of this phenotype found it to be accompanied by autophagic vesicle induction.

Hyperlipidemia and LDL cholesterol deposition in the subendothelial vessel space critically drive vascular disease [30]. Early on macrophage phagocytosis copes with the excess lipid, but as plaques enlarge, cell death in the core can leave acellular atherothrombotic material rich in cholesterol crystals [22]. Core necrosis, along with cap and shoulder thinning, makes such plaques hemodynamically vulnerable to tear spontaneously, or when disturbed by surgical interventions [31]. This dislodges plaque material that transports to systemic vascular beds causing stroke and tissue ischemia. By imaging plaques across three organismal settings we detected a variety of crystalline morphologies. In the macaque model the morphology distribution was needle structure dominated, whereas in both the human autopsy samples and in the *Ldlr*^*-/-*^ mice a more aggregated morphology dominated with occasional cuboidal features present. The discrete needle type of the Macaque samples allowed us to process the SHG images by Otsu’s method. We counted nearly one thousand needle shaped objects with the SIV^+^ samples being found to have a greater density of shorter needles per unit area. This did not translate into an overall increase in crystalline burden because by fluorescent reflectance imaging we did not detect a significant change in total crystalline burden. However, as reported by Baumer et. al., we did find evidence of cholesterol crystals in the endothelial layer and an example of the endothelia luminal surface pierced by a needle [32]. Of these images, the macrophage traversing an acellular field of cholesterol crystals attempting to phagocytose one of the needles was most interesting in that it appeared to capture a cellular process that has been linked to NLRP3 inflammasome activation, IL-1β secretion and CVD risk [18–20].

Because SRS imaging produces contrast by exciting endogenous molecular bond vibrations it can distinguish broad classes of biomolecules including nucleic acids, proteins and lipids [33]. Moreover, with hyperspectral scans this method can detect more nuanced chemical features of a class [34]. The spectral data cubes of the plaques from monkey, man and mice indicated a cholesterol ester dominant composition of both the needle and plate crystal morphologies. Such esterified cholesterol accumulates in macrophage foam cells that populate the subendothelial space early in the evolution of an atherosclerotic plaque. The cholesterol esters are stored in lipid droplets, but if storage capacity is compromised, non-esterified free cholesterol levels rise and can trigger apoptotic cell death [31]. Free cholesterol can also crystalize, and in a nephrectomized *ApoE*^*-/-*^ mouse model fed a high fat diet for 16 weeks, SRS imaging detected more crystalline material with a free-cholesterol dominated signature [26]. Why *Ldlr*^*-/-*^ mice fed the same diet but for a shorter 8-week period, and with full kidney function, have esterified cholesterol dominant crystals will need further investigation. However, because we are interested in the systemic effects of diets high in fat and refined sugar, we analyzed bone marrow derived macrophages from the *Ldlr*^*-/-*^ mice fed a high fat diet or not. At 4 weeks of feeding, occasional lipid engorged macrophages were observed and the phenotype became more prominent after 8 weeks of feeding. This observation is consistent with previous data showing that short periods on this western diet can alter the epigenetic and functional activity of the myeloid linage within the bone marrow niche [18].

We modeled these observations in macrophage J774A.1 cells fed either crystalline cholesterol, or acetylated LDL. Both treatments strongly induced auto-fluorescent puncta similar to those seen in the primary cultures of the BMDMs. Because the BMDM puncta did not strongly overlap with the lysosome compartment, we stained for lipid droplets with Nile-red in the J774A.1 model. But again, the puncta appeared to be a distinct structure. However, when co-stained for the LC3 marker of autophagosomes, we observed co-localization with the AcLDL generated puncta. Autophagy is a cellular process that is induced during time of cell stress, particularly when larger dysfunctional subcellular organelles such as mitochondria need to be broken down and their constituents recycled into other cellular processes and organelles. Inhibition of autophagic factors, such as ATG5, increases atherosclerosis in hypercholesterolemic mice, and excess dietary protein in these models has recently been found to exacerbate atherosclerosis by a mTOR dependent inhibition of autophagy [35, 36]. In aggregate, our observations are consistent with these findings in that crystalline cholesterol or acetylated LDL both strongly induced the LC3B associated auto-fluorescent puncta. These puncta were observed in macrophages derived from the bone marrow compartment of mice fed a high fat diet for as little as four weeks, suggesting that autophagic activation may be an early systemic stress response to consumption of calorically rich diets.

In summary, we found that atheroma from man, monkey and mice had cholesterol crystalline material with a hyperspectral signal dominated by cholesterol-esters. Both classic needle shaped crystals, as in the macaque model, or the more aggregated material with occasional cuboidal features found in the human and mouse samples showed the CE dominate signature. These findings suggest that in both early plaques, such as those from the mice, or more advanced plaques of the macaque and human samples with extensive acellular cores, esterified-cholesterol contributes significantly to the load of plaque crystal burden. However, SIV infection in macaques did not markedly change overall crystalline burden, and further study will be needed to ascertain whether infection may change the distribution of crystal morphologies, as suggested by the decrement in crystal length we found in SIV^+^ plaques.

## Supporting information

S1 Fig Cholesterol crystal burden in control and SIV+ macaques as measured by confocal reflectance microscopy. Frozen sections were visualized for cellularity by staining nuclei with DAPI and macrophages with the anti-F480 antibody, and crystal burden by reflectance. Plaque volume was calculated and the cholesterol crystal reflectance signal was expressed as a percent of plaque volume. Samples from 4 controls and 7 SIV+ were analyzed, a paired t-test = 0.85 showed no significant difference in crystal burden.
(TIF)
