# Supplementary figures and images for: Non-linear optical imaging of atherosclerotic plaques in the context of SIV and HIV infection prominently detects crystalline cholesterol esters
Source: PLoS One. 2021 May 13;16(5):e0251599. doi: 10.1371/journal.pone.0251599 (PMC8118308; doi:10.1371/journal.pone.0251599)

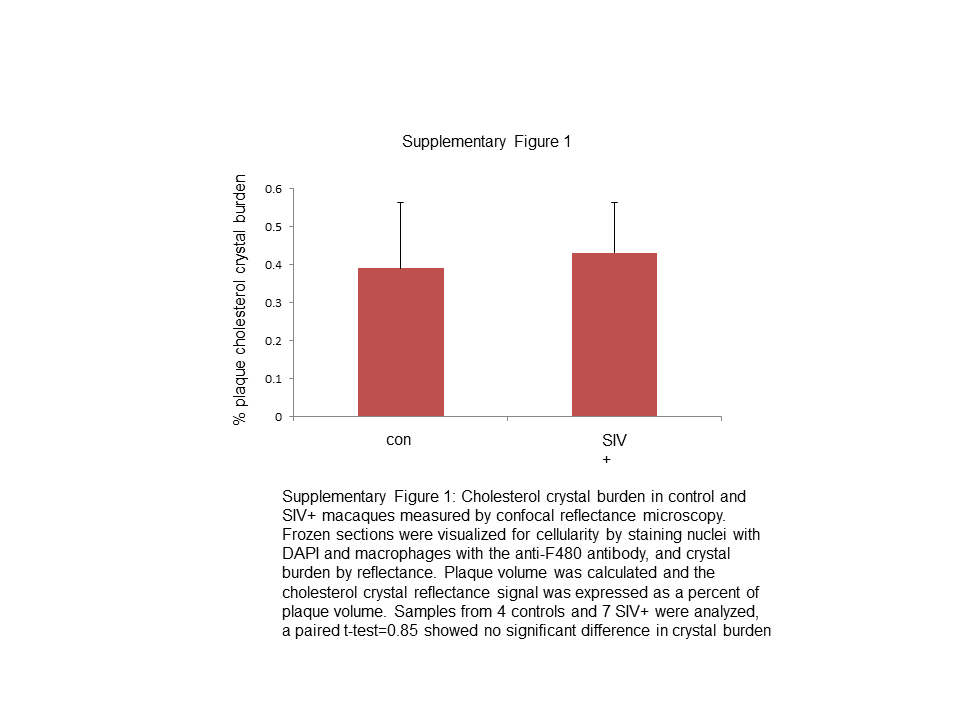

Supplement: S1 Fig — Frozen sections were visualized for cellularity by staining nuclei with DAPI and macrophages with the anti-F480 antibody, and crystal burden by reflectance. Plaque volume was calculated and the cholesterol crystal reflectance signal was expressed as a percent of plaque volume. Samples from 4 controls and 7 SIV+ were analyzed, a paired t-test = 0.85 showed no significant difference in crystal burden. (TIF) [file pone.0251599.s001.tif]
